# Effects of *Olea oleaster* leaf extract and purified oleuropein on ethanol-induced gastric ulcer in male Wistar rats

**DOI:** 10.22038/IJBMS.2024.76135.16474

**Published:** 2024

**Authors:** Fatiha Abdellah, Tayeb Silarbi, Ferjeni Zouidi, Khaled Hamden

**Affiliations:** 1 Laboratory of Bioresources, Integrative Biology and Exploiting, Higher Institute of Biotechnology of Monastir, University of Monastir, Tunisia; 2 Laboratory of Research on Local Animal Products, Ibn-Khaldoun University, Tiaret, Algeria; 3 Biology Department, Faculty of Sciences and Arts of Muhayil Asir, King Khaled University, Saudi Arabia

**Keywords:** Inflammation, Nitric oxide, Olea oleaster, Oleuropein, Ulcer

## Abstract

**Objective(s):**

Evaluating the effect of fresh Oleaster leaf extract (OLE) and purified oleuropein (OLR) on ethanol-induced gastric ulcers in rats. HPLC analysis demonstrates the presence of various polyphenol compounds such as ligstroside, luteolin derivative, oleuropein, and comselogoside.

**Materials and Methods:**

Gastric ulcer was induced by administration of ethanol by the gastric gavage route. The olive leaf extract was analyzed by HPLC-PDA-ESI-MS, and OLR was purified. These two compounds were given 2 hr before gastric ulcer induction by ethanol.

**Results:**

This study verified that OLE and purified OLR protect from ethanol-induced gastric ulceration and damage, evidenced by the significant decrease in gastric ulcer urea (by 74 and 58% respectively) and stomach mucus content (by 169 and 87% respectively). In addition, the ulcer index (UI) and curative index (CI) levels in the stomach of the rats treated with this supplement were also suppressed by 55 and 46%, respectively. OLE and OLR also decreased the gastric myeloperoxidase (MPO) activity and ameliorated the nitric oxide (NO) content. OLE and OL also ingestion suppressed gastric tumor necrosis factor-alpha (TNF-α) and interleukin (IL-6) rates. Macroscopic and histological findings revealed that OLE and OLR protect from gastric hemorrhage, severe disruption of the gastric mucosa, and neutrophil infiltration.

**Conclusion:**

Overall, the findings demonstrate that OLE and OLR have both promising potential with regard to the inhibition of gastric hemorrhage and lesions.

## Introduction

Gastric ulcer affects approximately 10% of people, and cause various digestive perturbations (1). Several factors favor the appearance of ulcers and gastric diseases such as alcohol, *Helicobacter pylori*, anti-inflammatory drugs, and caffeine (2). Too much alcohol ingestion caused an increase in inflammatory reactions and excessive production of mediators of inflammatory reactions such as interleukins and cytokines, and consequently gastric damage and permeability, and mucosal damage (3). The activation enzymes linked to gastric inflammation, such as MPO, and pro-inflammatory mediators, such as IL-6 and TNF-α, mediated the gastric mucosa injury (4-6). Additionally, NO was one of the major defensive systems in gastric mucosa defense and protection (7). Currently, commercial drugs have high efficiency for ulcer therapy such as H2-blockers and proton pump inhibitors which provide mucosal defense (8); however, they may cause many toxic effects (9). Therefore, one of the strategies for ulcer prevention and therapy is to search the nontoxic natural compounds. Much research is focused on investigating new natural drugs with greater safety and efficacy (10-14). Natural products, especially medicinal plants, have been an important source of functional substances for the prevention and treatment of various diseases and perturbations (15-19). Among these plants, *Olea europea* (Olive tree) is a natural source of various drugs such as acids, polyphenols, flavonoids, and fatty acids which are used for the treatment and prevention of many perturbations and pathology.

The Oleaster (Zebouj) is ever-present in Algeria and Tunisia. Oleaster tree leaves have been widely used in the prevention and treatment of various perturbations and diseases due to the richness of their leaves in several active phenolic compounds such as oleuropein (OLR) and hydroxytyrosol and many other polyphenolic drugs (20-24). No previous studies have evaluated the effect of OLE and OLR from *Olea europaea* var. *sylvestris* on ethanol-induced gastric MPO activity and NO, IL-6, and TNF-α level perturbation. Accordingly, the goal of this investigation is to evaluate the effect of OLE and purified OLR on ethanol-induced macroscopic and microscopic damage and ulceration in gastric tissues.

## Materials and Methods

***Plant material***

The wild olive tree (*O. europaea* var. *sylvestris*) was harvested from northern Algeria in January 2023 (Bordj-Ménaïel city: Kabylie, Algeria).

***Oleuropein extraction and purification***

Fresh oleaster leaves underwent air drying in the shade, followed by grinding with a coffee grinder to produce a dry powder. A total of 300 g of this powder was subjected to extraction with ethanol (1000 ml) under agitation for 24 hr and subsequently filtered. The resulting ethanol extract was stored at -20 ^°^C until analysis. An aliquot of 4 g from the dry leaf extract underwent chromatography on a silica gel column (480 cm), employing a solvent gradient of hexane, ethyl acetate, and methanol to purify OLR. Silica gel plates were used for TLC analysis (Merck, 60 F-254) to monitor chromatographic behavior, leading to the collection of nine homogeneous fraction groups. The third group was isolated through prep.TLC, eluted with a chloroform: ethanol mixture (8:2, V/V), yielding a single pure chemical with Rf=0.6. The presence of OLR was confirmed, and its quantity was determined using high-performance liquid chromatography (HPLC) set at 254 nm.

***HPLC-PDA-ESI(-)/MS analysis***

The HPLC-PDA-ESI(-)/MS analysis was conducted using a Surveyor LC system equipped with a diode array detector (Thermo Fisher, San Jose, CA, USA) and a Kinetex EVO C18 column (Phenomenex). The flow rate was set at 1 ml/min, and UV detection was performed at 254, 280, and 360 nm. The gradient program consisted of solvent A (0.01% formic acid) and solvent B (CH3CN), with a gradient sequence of 5 min at 10% B, a transition from 10% to 100% B over 42 min, a 5 min hold at 100% B, a return to 10% B in 3 min, and a final 5 min at 10% B. A 4 μl injection volume was utilized. Following flow splitting, the LC system was linked to an LXQ linear ion trap (Thermo Fisher). Operational conditions for (-) ESI mode were optimized, including a sheath gas flow of 50 arbitrary units, an auxiliary gas flow of 8 arbitrary units, a spray voltage of +4.0 kV, an ion transfer tube temperature of 375 ^°^C, and a capillary voltage of 35 V. Mass spectral data were recorded through full scanning in the m/z range of 100–1800. MSn experiments employed a 2 Da isolation width and a normalized collision energy of 35%. Xcalibur software, version 2.0 (Thermo Fisher) was used for data recording and processing. Metabolite identification relied on a comparison of their λmax, retention times, and MS data with references from previous studies, as detailed in the results and discussions section. The extraction of OLR from Olea oleaster leaves followed a protocol outlined in a prior study and utilized an HPLC Agilent 1260 (Agilent Technologies, CA, USA) (25).

***Anti-ulcer activity evaluation***

*Animal model*

25 Male *Wistar* rats weighing 187±11 g and aged 6 weeks were housed under a controlled environment and permitted water and food pellets *ad libitum*. The rats were divided into four groups, five rats in each group. Control rats received distilled water at a dose of 0.5 ml/100g bw (C). Gastric ulcer rats received absolute ethanol at dose (0.5 ml/100 g bw) by gastric gavage route (26) and named (U). U+OLE, U+OL, and U+OMP: gastric ulcerated rats received 200 mg/kg bw of OLE or OL (27), or 30 mg omeprazole (OMP)(28) as a synthetic proton pump inhibitor drug (29). Ingestion of ethanol was supplemented by gastric gavage route to rats, 2 hr after drug ingestion. Two hours later, the rats were put to sleep for two to five minutes in a small room using a cotton ball soaked in 1.9% diethyl ether, sacrificed by decapitation and the stomach was detached from rats and was opened along the greater curvature. The stomachs were gently rinsed with iced cold phosphate buffer to clean. Cleaned stomachs were photographed and photos were taken to examine the gastric lesions. Stomach fragments were embedded in paraffin wax and treated in 10% formaldehyde for histological analysis (30). At last, histological slices measuring 5 µm in thickness were cut from the stomachs were captured using an Olympus CX41 light microscope after being stained with hematoxylin and eosin solution (H&E). Parts of the stomachs of the different groups of rats were crushed in phosphate buffer, after centrifugation at 5000 rpm, and the samples were stored at -80 ^°^C until use for biochemical analysis. The area of gastric lesion was measured using the Image J program.

*Ethical permission*

Institutional Animal Ethics Committee (ISBM, UM, Monastir) approval (No. Code: 86/609/EEC dated 03.11.2022) was obtained before the beginning of this rat experimentation.

*Gastric ulcer index determination and biochemical analysis*

The total ulcer area for the cleansed stomach was measured using an inverted microscope with a digital camera. The stomach ulcer area was calculated by the Image J software (version 1.51J8) with digital calculable distance (mm) using an e-ruler. The ulcer index (UI) was determined following the formula: Ulcer index=[(Ulcer area)/(Total mucosa surface area)]×100. The curative ratio was determined following the formula: Curative ratio=(US ulcerated-US treated)/(US control)(31). The gastric MPO activity was determined by the modified method described by Bradley *et al.* (32) by spectrophotometric methods through determination of the absorbance at 460 nm using hydrogen peroxide as substrate. The gastric NO rate was determined by the protocol described previously (33). Gastric TNF-α and IL-6 rates were calculated by enzyme-linked immunosorbent a (Kit-1015901 and kit-67768; Shanghai MLBIO Biotechnology Co., Ltd., Shanghai, China). 

***Statistical analysis***

The curves of this study are presented as MEAN±SEM. The variations were calculated by one-way analysis of variance (ANOVA) followed by the Fisher test (Stat View). 

## Results

***HPLC-PDA-ESI(-)/MS analysis***

**Figure 1:**
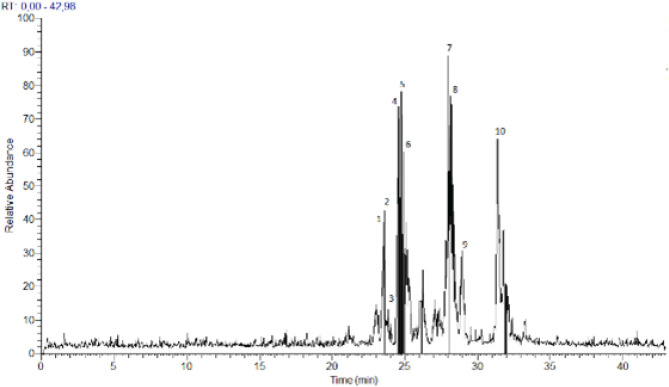
HPLC-PDA-ESI(-)/MS Chromatographic profile of ethanolic extract from Oleaster leaves

The composition of phenolics in the ethanolic extract derived from Oleaster leaves was determined through HPLC-PDA-ESI-MS. Metabolite profiling using HRMS was conducted in negative mode (see Figure 1). The identified compounds are listed in Table 1, along with their retention time (Rt) in minutes, monoisotopic mass of the pseudomolecular ion (m/z), elemental composition (EC) [M-H]-1, RDBeq values, and their primary HRMS/MS fragments. A total of 10 phenolic compounds belonging to two chemical classes, secoiridoids and flavonoids, were identified in the Oleaster extract. Secoiridoids constituted the primary compounds found in Oleaster leaves. For instance, Compound 1, eluting at Rt=23.61 min, exhibited a pseudo-molecular ion [M-H]- at 715.2448, identified as methoxynuzhenide. Its MS2 spectrum displayed a fragment ion at m/z 553.1707 (C_26_H_33_O_13_), likely due to the loss of a glucose unit, along with base peaks at m/z 323.0759 (C_15_H_15_O_8_), 285.0397 (C_15_H_9_O_6_), and 299.0546 (C_16_H_11_O_6_). Compound 3 displayed a precursor ion [M-H]- at m/z 543.2000, consistent with the formula C_25_H_35_O_13_. Compound 4, detected at Rt=24.72 min, yielded a deprotonated molecule [M-H]- at m/z 535.2667, identified as Comselogoside (p-Coumaroyl-6-secologanoside) based on HRMS and literature data. Compound 5, eluting at Rt=24.94 min, exhibited a quasi-molecular ion at m/z 701.2293. Compound 6, with a precursor ion [M-H]- at m/z 685.2336, was detected at Rt=24.96 min. Compound 7, eluting at Rt=28.01 min, presented a deprotonated ion [M-H]- at m/z 539.1333, tentatively assigned as OLR, previously identified in various organs of two *O. europaea* varieties. Its MS2 spectrum displayed main fragment ions at m/z 377.0281 (C_19_H_21_O_8_), 307.1074 (C_15_H_15_O_7_), 345.0282 (C_18_H_17_O_7_), and 275.0410 (C_15_H_15_O_5_). Compound 8, detected at Rt=28.38 min, also exhibited the deprotonated molecule [M-H]- at m/z 539.0637, shared similar fragmentation patterns with m/z 377, 307, and 275, similar to OLR. Compound 9, eluting at Rt=29.02 min, was detected with a precursor ion [M-H]- at m/z 403.2000. Compound 10, with Rt=31.39 min, possessed a pseudo-molecular ion [M-H]- at m/z 523.1333.

***Effect of OLE and OLR on ulcer area, gastric mucus rate, ulcer index, and curative index***

**Figure 2:**
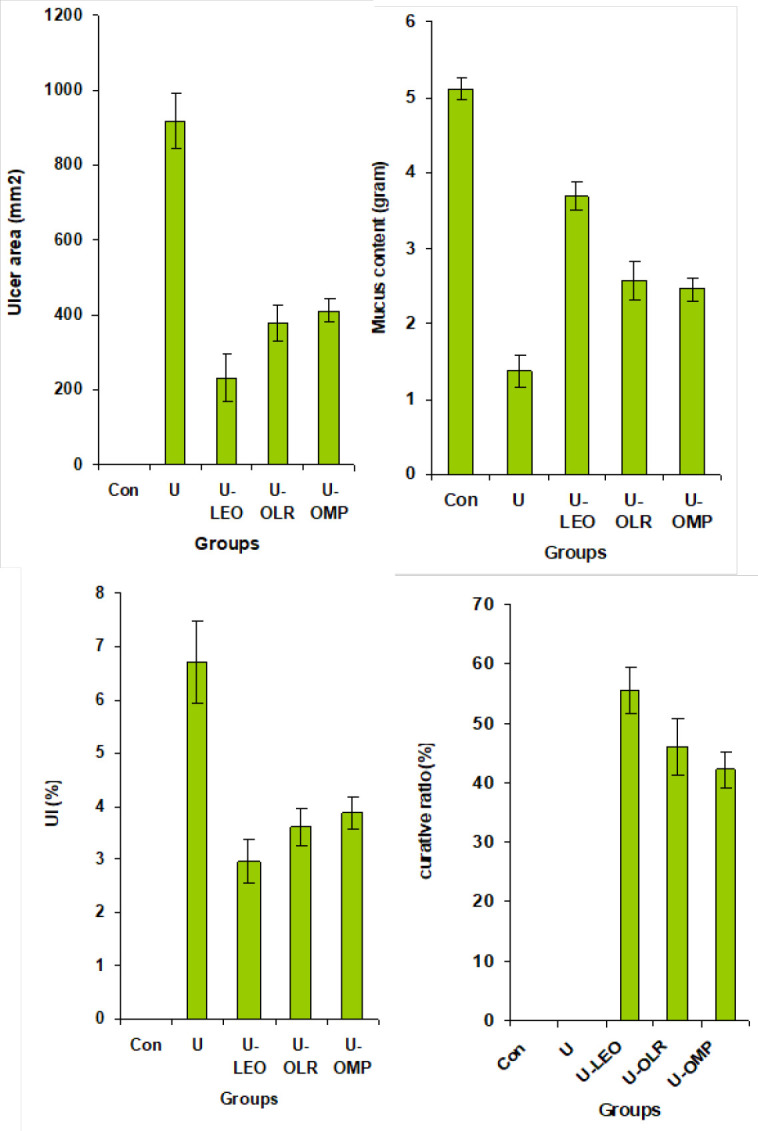
Ulcer area, gastric mucus, UI and CI levels in OLE and OLR-ulcered rats

This study showed that OLE and OLR ingestion protects from ethanol-provoked gastric damage and ulceration. Administration of OLE and OLR exerts a protective action against gastric macroscopic injury, which was shown by the presence of hemorrhage evidenced by a significant decrease in gastric ulcer area by 74 and 58% and ulcer index by 55 and 46%, respectively. In addition, OLE and OLR stimulate the gastric mucus content by 169 and 87% and increase the curative index by 55 and 46%, respectively as compared to untreated gastric-ulcer rats (Figure 2). 

***Effect of OLE and OLR on gastric mucosal MPO activity and NO rate***

**Figure 3:**
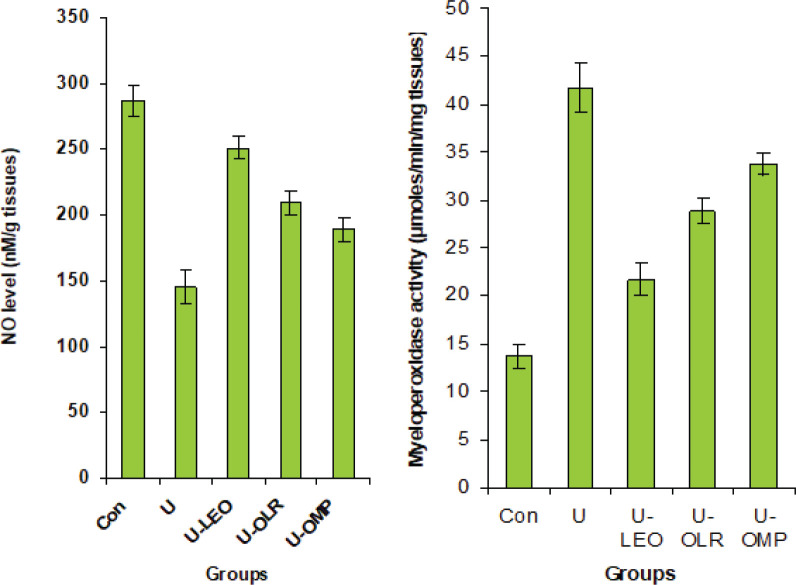
Gastric MPO activity and NO rate in OLE and OLR gastric ulceration treated rats

As shown in Figure 3, gastric ulcer rats had increased MPO activity in the stomach by 204%. Conversely, NO was significantly decreased by 36.9% as compared to the normal group. However, in ulcer-rat treated with OLE and OLR, a significant decrease in gastric MPO activity (by 47 and 30%, respectively) and an increase in NO rate (by 38 and 33%, respectively)was observed. 

***Effect of OLE and OLR on gastric IL6 and TNF-α levels***

**Figure 4:**
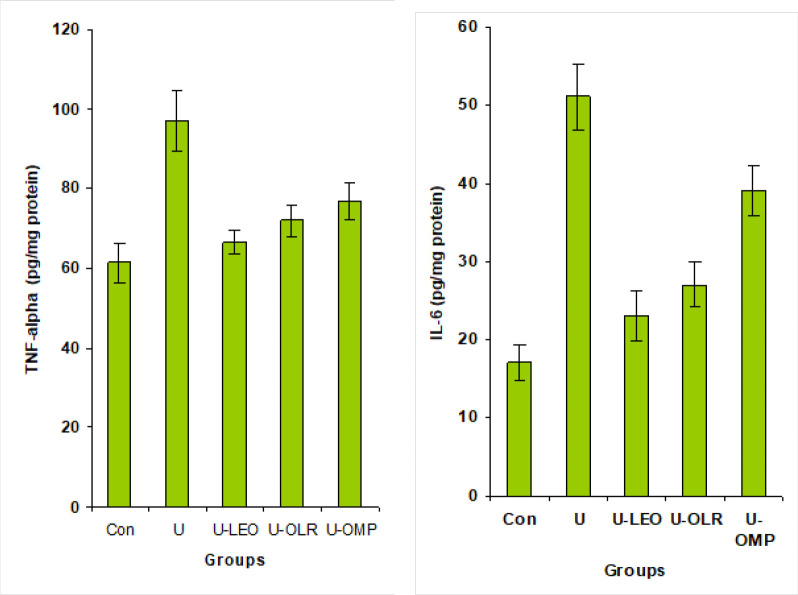
Gastric TNF-α and IL-6 levels in OLE and OLR gastric ulceration treated rats

As shown in Figure 4, ethanol ingestion caused a significant increase in gastric TNF-α and IL-6 by 58 and 200%, respectively as compared with the normal rats. However, the administration of OLE and OLR to gastric-ulcer rats protected from inflammation in gastric tissues and showed a potential decrease of TNF-α (by 21 and 17%, respectively) and IL-6 (54 and 47%, respectively) as compared to untreated gastric-ulcer rat.

***Effect of OLE and OLR on macroscopic gastric damage***

**Figure 5:**
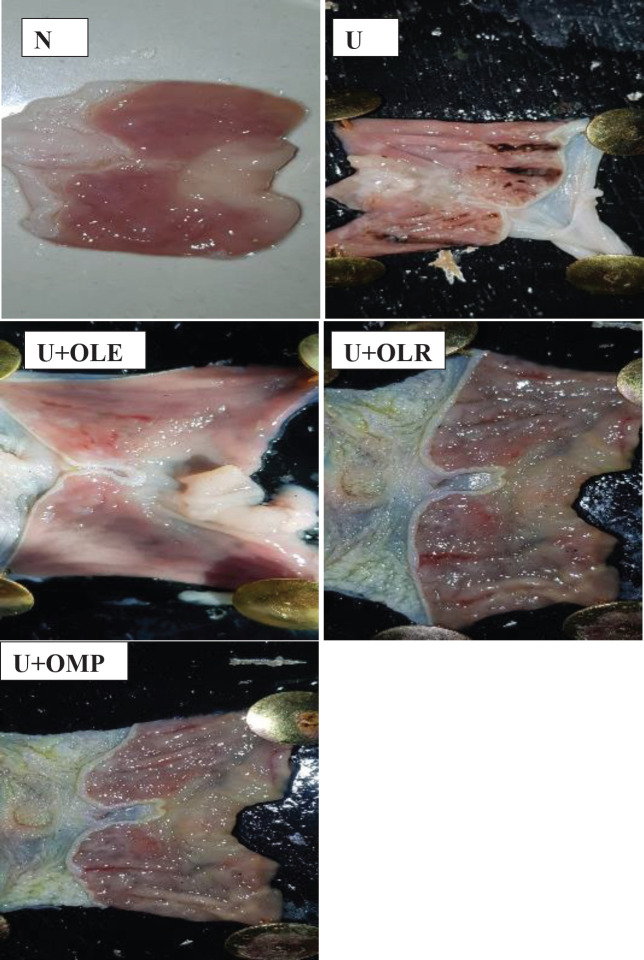
Photos from the pathology slides showing the effect of supplementation of OLE and OLR to ethanol-induced gastric ulcer rats

Our results show that the ingestion of ethanol causes severe gastric hemorrhage or even ulceration, this is evidenced by macroscopic observations (Figure 5). On the contrary, pretreatment of rats with omeprazole, OLE, or OLR protects effectively against gastric hemorrhage and ulceration.

***Effect of OLE and OLR on histological evaluation of gastric damage***

**Figure 6:**
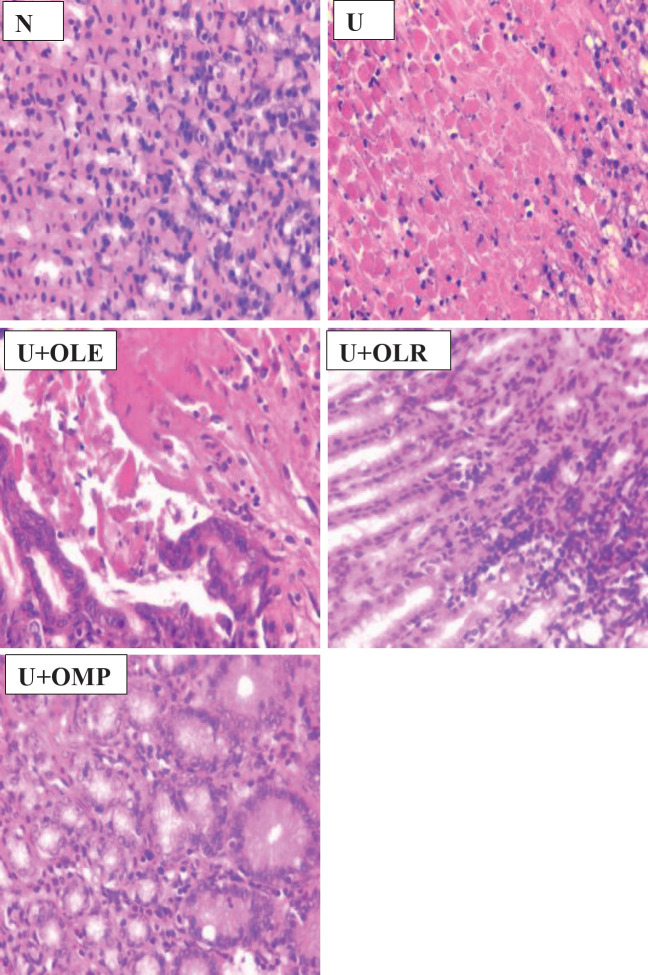
Photos from the pathology slides showing the effect of administration of OLE or OLR to gastric mucosa ulcers in rats

In comparison to the gastric mucosa of normal rats, this study demonstrated that supplementing ethanol by the gastric gavage route caused a variety of lesions in the stomach of rats, including severe disruption of the gastric mucosa, flattening of the gastric mucosa, and necrotic lesions that deeply penetrated the mucosa and sub-mucosal layers (Figure 6). Furthermore, we demonstrated that omeprazole, OLE, or OLR prevented gastrointestinal mucosal ulcers, flattened gastric mucosa, and necrotic lesions in rats’ stomachs.

**Table 1:** HPLC-PDA-ESI(-)/MS analysis of secondary metabolites identified in the ethanolic extract from Oleaster leaves

**N°**	**Compounds**	**Rt (min)**	**Measured m/z**	**EC[M-H]** ^-^	**RDBeq** **values**	**Main fragments** **(EC, RDBeq)**
**1**	methoxynuzhenide	23.61	715.3333	C_32_H_43_O_18_	11.5	553.1707 (C_26_H_33_O_13_. 10.5)
**2**	Luteolin derivative	23.64	623.2667	C_31_H_27_O_14_	18.5	323.0759 (C_15_H_15_O_8_. 8.5) 285.0397 (C_15_H_9_O_6_. 11.5) 299.0546 (C_16_H_11_O_6_. 11.5)
**3**	Dihydro oleuropein	23.87	543.2000	C_25_H_35_O_13_	8.5	525.1980 (C_25_H_33_O_2_. 9.5) 513.1982 (C_24_H_33_O_12_. 8.5)
**4**	Comselogoside (p-Coumaroyl-6-secologanoside)	24.72	535.2667	C_25_H_27_O_13_	12.5	491.1543 (C_24_H_27_O_11_. 11.5)
**5**	Oleuropein glucoside or neo-nuzhenide	24.94	701.2293	C_31_H_41_O_18_	11.5	539.1756 (C_25_H_31_O_13_. 10.5) 377.1226 (C_19_H_21_O_8_. 9.5) 307.0786 (C_15_H_15_O_7_. 8.5)
**6**	nuzhenide	24.96	685.2336	C_31_H_41_O_17_	11.5	523.1809 (C_25_H_31_O_12_.10.5)
**7**	Oleuropein	28.01	539.1333	C_25_H_31_O_13_	10.5	377.0281 (C_19_H_21_O_8_^.^ 9.5) 307.1074 (C_15_H_15_O_7_. 8.5) 345.0282 (C_18_H_17_O_7_. 10.5) 275.0410 (C_15_H_15_O_5_. 8.5)
**8**	Oleuroside	28.38	539.0637	C_25_H_31_O_13_	10.5	307.0813 (C_15_H_15_O_7_. 8.5) 377.1228 (C_19_H_21_O_8_. 9.5) 275.0410 (C_15_H_15_O_5_. 8.5)
**9**	Oleoside methyl ester	29.02	403. 2000	C_17_H_23_O_11_	6.5	223.0601(C_11_H_11_O_5_. 6.5) 179.0555 (C_6_H_11_O_6_. 1.5)
**10**	Ligstroside	31.39	523.1333	C_25_H_31_O_12_	10.5	361.1288 (C_19_H_21_O_7_. 9.5) 291.0869(C_15_H_15_O_6_. 8.5) 259.0932(C_15_H_15_O_4_. 8.5)

Each value represents the mean±SEM for each group (n=5). The statistical analysis is presented as follows: *P<0.05 vs normal rats; ^#^*P<*0.05 vs ethanol-induced gastric ulcer in rats. ^@^*P<*0.05 vs ethanol-induced gastric ulcer in rats treated with OLE; ^&^*P<*0.05 vs ethanol-induced gastric ulcer in rats. ^@^*P<*0.05 vs ethanol-induced gastric ulcer in rats treated with OLR

UI: ulcer index; CI: curative index; OLE: Oleaster leaf extract; OLR: Oleuropein

Each value represents the mean±SEM for each group (n=5). The statistical analysis is presented as follows: *P<0.05 vs normal rats; ^#^*P<*0.05 vs ethanol-induced gastric ulcer in rats; ^@^*P<*0.05 vs ethanol-induced gastric ulcer in rats treated with OLE; ^&^*P<*0.05 vs ethanol-induced gastric ulcer in rats. ^@^*P<*0.05 vs ethanol-induced gastric ulcer in rats treated with OLR

MPO: myeloperoxidase; NO: Nitric oxide; OLE: Oleaster leaf extract; OLR: Oleuropein; OMP: Omeprazole

Each value represents the mean±SEM for each group (n=5). The statistical analysis is presented as follows: *P<0.05 vs normal rats; ^#^*P<*0.05 vs ethanol-induced gastric ulcer in rats. ^@^*P<*0.05 vs ethanol-induced gastric ulcer in rats treated with OLE; ^&^*P<*0.05 vs ethanol-induced gastric ulcer in rats. ^@^*P<*0.05 vs ethanol-induced gastric ulcer in rats treated with OLR

OLE: Oleaster leaf extract; OLR: Oleuropein

N: appearance normal of gastric tissues without ulcer; U: gastric lesion and ulcerations with hemorrhagic regions. U+OLE, U+OLR, and U+OMP: Gastric ulcerated rats treated with OLE or OLR or at a dose of 200 mg/kg bw (U+OLE and U+OLR), a protective effect from gastric injury and hemorrhagic was observed, and only gastric mucosa are much milder compared to the injuries seen in the ulcer control rats. In Omeprazole ulcer-gastric rats (U+OMP); a partial protective action from gastric lesions and ulcerations with hemorrhagic regions was shown, and this group presents few and middle hemorrhagic regions

OLE: Oleaster leaf extract; OLR: Oleuropein; OMP: Omeprazole

There is severe disruption of the gastric mucosa, necrotic lesions penetrating deeply into the mucosa, and neutrophil infiltration in ulcer rats as compared to the gastric mucosa of normal rats (H&E stain,400X). U+800 mg BRE from pulp and U+800 mg BRE from peel: stomach histological section of rat treated with pulp BRE at dose of 800 mg/kg bw: the gastric wall appears normally, but there are a few small lesions in gastric mucosa. Stomach histological section of the rat treated with OmepZ at dose of 30 mg/kg bw: mild disruption to the submucosal layer

OLE: Oleaster leaf extract; OLR: Oleuropein; OMP: Omeprazole

**Figure 7:**
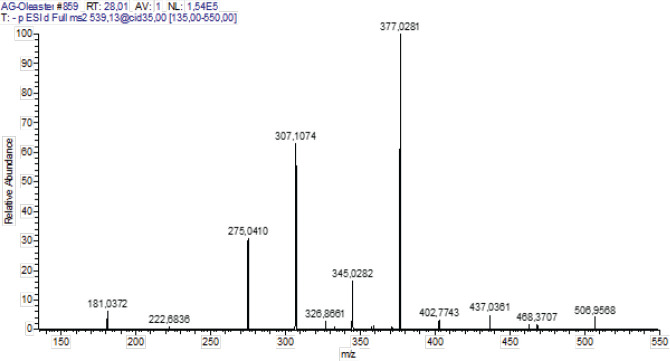
ESI(-)/MS2 specrtum of Oleuropein (m/z 539.1333)

## Discussion

Olea is a plant whose culinary uses have been extensively researched; a significant portion of the global population consumes both oil and fruits daily. Olives are a staple food in the Mediterranean diet. Olive polyphenols have long been safely consumed by humans, as evidenced by the usage of olive fruits and oil in the diet without any known negative consequences. In olive-producing countries, olive leaves have also traditionally been fed to animals and consumed by people for health reasons (34, 35). Olive leaves’ phenolic chemicals are thought to be responsible for these qualities. OLR, the most common polyphenol in olive leaves, has antiviral properties, protects the membrane from lipid oxidation, enhances lipid metabolism, bolsters coronary blood vessel dilatation, acts as an antiarrhythmic, protects from inflammation, prevents hypertensive cell death in cancer patients, and has therapeutic and preventative effects against several other disorders (36). This study showed that OLE contents of 10 phenolic compounds were identified in the Oleaster extract belonging to two chemical classes: secoiridoids and flavonoids. Secoiridoids were the main compounds found in Oleaster leaves. Among them, OLR (Rt 28.01 min) was the major phenolic component in leaf ethanolic extract. It was already described as the most representative secondary metabolite of cultivated olive leaves (37, 38). Compound 1, with retention time Rt=23.61 min, has a pseudo-molecular ion [M-H]- at 715.2448. This compound was assigned as methoxynuzhenide. Its MS2 spectrum shows the fragment ion at m/z 553.1707 (C_26_H_33_O_13_). It was previously identified in other varieties of *O. europaea* by Baccouri *et al*. (39). Compound 2 with Rt=23.64 min gave a molecular ion [M-H]- at m/z 623.2667. Based on literature this compound was identified as a luteolin derivative (40). Its MS2 spectrum contained a base peak at m/z 323.0759 (C_15_H_15_O_8_) followed by 285.0397 (C_15_H_9_O_6_) and 299.0546 (C_16_H_11_O_6_). Compound 3 presents a precursor ion [M-H]- at m/z 543.2000 consistent with the formula C_25_H_35_O_13_. This compound was assigned as Dihydro oleuropein by comparison to literature data (41, 42). Compound 4 was detected at a retention time Rt=24.72 min. This compound yielded the deprotonated molecule [M-H]- at m/z 535.2667. Based on HRMS and literature data, it was identified as Comselogoside (p-Coumaroyl-6-secologanoside)(43). Its fragment at m/z 491.1543 (C_24_H_27_O_11_) resulted from a loss of CO_2_, which is in good concordance with the literature data (44, 45). Compound 5 (Rt=24.94 min) possesses a quasi-molecular ion at m/z 701.2293. It was tentatively identified as Oleuropein glucoside or neo-nuzhenide (46). Compound 6 with a precursor ion [M-H]- at m/z 685.2336 was detected at a retention time of 24.96 min. It was identified as nuzhenide (47). Compound 7 detected at Rt=28.01 presented a deprotonated ion [M-H]- at m/z 539.1333. This compound tentatively assigned as OLR was previously identified in different organs of two varieties of *O. europaea* (Koroneiki and Chetoui) by Toumi *et al*., (48). Its MS2 spectrum (Figure 7) shows the main fragments ions at m/z 377.0281 (C_19_H_21_O_8_), 307.1074 (C_15_H_15_O_7_), 345.0282 (C_18_H_17_O_7_) and 275.0410 (C_15_H_15_O_5_). Compound 8 was detected at a retention time of 28.38 min and presented the same deprotonated molecule [M-H]- at m/z 539.0637 of compound 7 (OLR). As described in literature (49, 50), this compound was identified as Oleuroside which differs from OLR in the position of a double bound in the elenolic acid moiety. It shares a similar fragmentation pattern with m/z 377, 307, and 275 to be the main product ions. Compound 9 with a retention time at Rt=29.02 min was detected with a precursor ion [M-H]- at m/z 403. 2000. It was conﬁrmed by comparison to authentic standards and consequently could correspond to Oleoside methyl ester. Its MS2 fragmentation indicated the presence of two ions at m/z 223.0601 (C_11_H_11_O_5_) and 179.0555 (C_6_H_11_O_6_). Compound 10 with retention time Rt=31.39 min possesses a pseudo-molecular ion [M-H]- at m/z 523.1333. In a previous report, researchers (51), were able to identify this compound as ligstroside. Its MS2 fragmentation indicated the existence of a base peak at m/z 361 (C_19_H_21_O_7_) followed by 291 (C_15_H_15_O_6_) and 259 (C_15_H_15_O_4_).

One of the strategies for the prevention and treatment of gastric ulcers is the inhibition of inflammation and leukocyte infiltration in gastric mucus and the suppression of inflammatory mediators in gastric tissues (52, 53). In this study, ingestion of ethanol caused inflammation in gastric mucosa, evidenced by a significant increase in the MPO activity and a decrease in NO rate. In addition, the presence of inflammation in the gastric mucus is confirmed by an elevated level of TNF-α and IL6. The gastric inflammation induced by ethanol caused damage and death of the gastric mucosa cells and consequently an important decrease in the gastric mucus by 73% as compared to normal rats. The damage to gastric mucus cells is also observed by visual morphological changes such as gastric lesions and ulcerations with hemorrhagic regions (Figure 5) and histological alteration as severe. There is severe disruption of the gastric mucosa and necrotic lesions penetrating deeply into mucosa and neutrophils infiltration in ulcer rats as compared to the gastric mucosa of normal rats (Figure 6), and subsequently a significant increase in UA and UI by 73 and about 70%, respectively as compared to normal rats. 

In ulcerated rats treated with OLE and OLR, an effective protective effect from gastric damage and ulceration has been observed. Pretreatment of ulcerated rats by OLE or OLR protected from gastric mucosa and necrotic lesions penetrating deeply into mucosa and neutrophils infiltration in ulcer rats as compared to the gastric mucosa of normal rats (Figure 6). In addition, ingestion of OLE or OLR by ulcerated rats protects and prevents severe disruptions of the gastric wall and gastric lesions and ulcerations with hemorrhagic regions in rats with ethanol-induced gastric ulcers (Figure 5). In addition, the supplementation of OLE or OLR to ethanol-induced gastric ulcer tats inhibits inflammation at the level of gastric tissues, observed by an important inhibition of MPO activity and TNFα and IL6 rates and improvement of NO rate. Researchers (54) demonstrated that pre-treatment with *Olea europea* L. cv. *Arbequina* extract before indomethacin administration potentially protects from microscopic and macroscopic gastric mucosal lesions and alteration. A study (55) revealed that the ingestion of raw olive leaf extract by ethanol-induced gastric ulcer rats prevents gastric hemorrhagic lesions, decreases ulcers, and reduces the inflammatory mediator’s levels. Prior studies show that olive leaf extract protects from various human diseases and functions in certain diseases (56). Rezagholizadeh *et al.* (57) have indicated that OLR possesses the ability to inhibit several inflammatory enzymes, notably lipoxygenases, and reduce the levels of pro-inflammatory cytokines such as IL-6, TNF-α, and IL-1β. OLR has also been shown to scavenge superoxide anions, block radicals produced from hypochlorous acid, and modify the MAPK signaling pathway. Alethari *et al.* (58) showed that ingestion of olive leaf extract protects against inflammation by stopping the increase in TNF-α and IL-6 levels. Mahmoud (59) found that olive leaf extract protects from mucosal injury, damage in surface mucosal cells, and destruction of areas of gastric ulcer in experimental rats (60). Moreover, olive leaves decrease mucosal height and reduce neutrophil infiltration during inflammation. The results of this study suggest that the anti-inflammatory effect of OLE may be due to the inhibition of prostaglandin biosynthesis. These results suggest that OLE contains some bioactive phytochemical compounds that exert an anti-inflammatory activity (61-63). Similarly, a study (64) showed that administration of OLR at a dose of 500 mg/kg daily alleviates gastric damage and ulceration by the decrease of inflammation as tumor TNF-α, prostaglandin E2, endothelial nitric oxide synthase, and caspase-3 levels in indomethacin-induced gastric ulcer rat. Abd-Allah* et al*. (65) also showed that OLR ingestion by acetic acid-induced ulcerative colitis rats causes a significant reduction in stress oxidants, regulates the pro-inflammatory cytokines rates, down-regulates Bax, and up-regulates Bcl2. Moreover, OLR exerts anti-inflammatory, antioxidant, and anti-apoptotic effects in the ulcerative colitis experimental model (66). 

## Conclusion

We conclude that OLE or OLR ingestion significantly protects from ethanol-induced gastric damage and ulceration. These anti-ulcer effects of Oleaster leaf extract and purified OLR are mediated by inhibition of inflammation and induction of NO levels in gastric tissues.

## Authors’ Contributions

F A and T S conceived and designed the experiments. F Z conceived and designed the experiments and analyzed and interpreted the data. K H performed the experiments, analyzed and interpreted the data, contributed reagents, materials, analysis tools, or data, and wrote the paper.

## Funding statement

This study did not receive any funding in any form.

## Data Availability

The data used to support the findings of this study are available upon request from the corresponding author.

## Conflicts of Interest

The authors declare no competing financial interest.
